# Realization of NIR-II 3D whole-body contour and tumor blood vessels imaging in small animals using rotational stereo vision technique

**DOI:** 10.1117/1.JBO.28.9.094807

**Published:** 2023-05-24

**Authors:** Shih-Po Su, Yun-Chen Lee, Syue-Liang Lin, Yi-Xuan Li, Min-Ying Lin, Yang-Hsiang Chan, Yi-Jang Lee, Muh-Hwa Yang, Huihua Kenny Chiang

**Affiliations:** a National Yang Ming Chiao Tung University, Department of Biomedical Engineering, Taipei, Taiwan; b National Yang Ming Chiao Tung University, Biomedical Engineering Research and Development Center, Taipei, Taiwan; c National Yang Ming Chiao Tung University, Department of Applied Chemistry, Hsinchu, Taiwan; d National Yang Ming Chiao Tung University, Department of Biomedical Imaging and Radiological Sciences, Taipei, Taiwan; e National Yang Ming Chiao Tung University, Institute of Clinical Medicine, Taipei, Taiwan

**Keywords:** second near-infrared, three-dimensional, rotational stereo imaging, polymer dots, blood vessels imaging, tumor imaging, contour

## Abstract

**Significance:**

Optical imaging in the second near-infrared (NIR-II, 1000 to 1700 nm) region is capable of deep tumor vascular imaging due to low light scattering and low autofluorescence. Non-invasive real-time NIR-II fluorescence imaging is instrumental in monitoring tumor status.

**Aim:**

Our aim is to develop an NIR-II fluorescence rotational stereo imaging system for 360-deg three-dimensional (3D) imaging of whole-body blood vessels, tumor vessels, and 3D contour of mice.

**Approach:**

Our study combined an NIR-II camera with a 360-deg rotational stereovision technique for tumor vascular imaging and 3D surface contour for mice. Moreover, self-made NIR-II fluorescent polymer dots were applied in high-contrast NIR-II vascular imaging, along with a 3D blood vessel enhancement algorithm for acquiring high-resolution 3D blood vessel images. The system was validated with a custom-made 3D printing phantom and *in vivo* experiments of 4T1 tumor-bearing mice.

**Results:**

The results showed that the NIR-II 3D 360-deg tumor blood vessels and mice contour could be reconstructed with 0.15 mm spatial resolution, 0.3 mm depth resolution, and 5 mm imaging depth in an *ex vivo* experiment.

**Conclusions:**

The pioneering development of an NIR-II 3D 360-deg rotational stereo imaging system was first applied in small animal tumor blood vessel imaging and 3D surface contour imaging, demonstrating its capability of reconstructing tumor blood vessels and mice contour. Therefore, the 3D imaging system can be instrumental in monitoring tumor therapy effects.

## 1 Introduction

Over the past decade, fluorescence molecular imaging systems have been extensively applied in monitoring or analyzing drug distribution in preclinical studies.^1^^–^^3^ In recent years, researchers have proven that fluorescence imaging in the second near-infrared window (NIR-II, 1000 to 1700 nm) enables deeper tissue penetration and higher spatial resolution than NIR-I (∼800 to 900 nm) fluorescence imaging due to less scattering of longer-wavelength photons and minimal autofluorescence background in this region.^4^^–^^6^ Consequently, NIR-II fluorescence imaging has been reported to produce high-resolution structural imaging on small animals, arousing substantial research interest.^7^^–^^9^ Several NIR-II small animal fluorescence imaging systems have been developed for various biomedical applications, such as cerebrovascular imaging,^10^ tumor targeting,^11^^,^^12^ and bone targeting imaging.^13^^,^^14^ Meanwhile, NIR-II fluorescent agents, such as semiconducting polymer nanoparticles, for imaging systems have been developed recently to visualize blood vessels and tumors via high fluorescence imaging signals in preclinical studies.^15^^–^^18^ Herein, our previous studies have synthesized NIR-II fluorescent polymer dots for NIR-II blood vessel imaging with a high signal-to-noise ratio (SNR).^19^^,^^20^

Non-invasive optical imaging techniques, such as fluorescence confocal imaging, photoacoustic imaging, and stereoscopic imaging techniques, can accomplish three-dimensional (3D) imaging. For *in vivo* research, the visualization of 3D vascular structure is conducive to understanding blood supply and evaluating tumor status.^21^ However, conventional optical 3D vascular imaging technologies have some drawbacks. For instance, despite its ability for high-resolution imaging of blood vessels, fluorescence confocal imaging has a limited imaging field, reaching only 2×2  mm in the case of NIR-II confocal microscopy.^22^^,^^23^ For its good resolution (∼25  μm), 3D NIR-II photoacoustic imaging needs expensive equipment, and scanning is time-consuming.^24^

The binocular stereo vision 3D-imaging method, via integration of object images from left- and right-eye views, has been developed over the past two decades, boasting the merits of no contact, low cost, and high precision.^25^^–^^27^ Many 3D surface imaging techniques have been developed, such as stereoscopic vision,^28^ structured light,^29^ and time-of-flight,^30^ which are capable of offering depth information on a scene and thus characterizing the spatial distribution of an object in full, such as blood vessel structures or tumor vessels. Later, the potential of NIR-I stereo imaging in visualizing 3D blood vessel structures *in vivo* has been demonstrated. Chen et al.^31^^,^^32^ developed a stereo system consisting of two digital NIR-I CCDs to visualize 3D blood vessel structures under the skin. However, the NIR-I window is flawed with optical diffusion, lower resolution, and limitation of tissue penetration to 3 mm in depth, problems that can be rectified with the substitution of the NIR-II window, boasting increased tissue penetration depth, and enhanced imaging contrast.^33^^,^^34^ Our previous study built a stereo NIR-II fluorescence imaging system with a planner moving plate for reconstructing 3D tumor vascular and blood vessel images with one NIR-II camera only.^35^ The system overcomes the problem of substantial background noise due to much lower scattering in the NIR-II region, but it is flawed with a limited view angle and the need for a large operating platform.

The paper puts forth an NIR-II 3D fluorescence rotational imaging system via a combination of an NIR-II camera and a 360-deg rotational stage, enabling the reconstruction of 360-deg 3D images on blood vessels and mouse contour with a spatial resolution of 0.15 mm, imaging depth of 5 mm, and depth resolution of 0.3 mm in an *ex vivo* experiment. This imaging system boasts a promising economical and user-friendly solution for small animal NIR-II 3D imaging.

## 2 Materials and Methods

### 2.1 Imaging System and Instrumentation

**Fig. 1:**
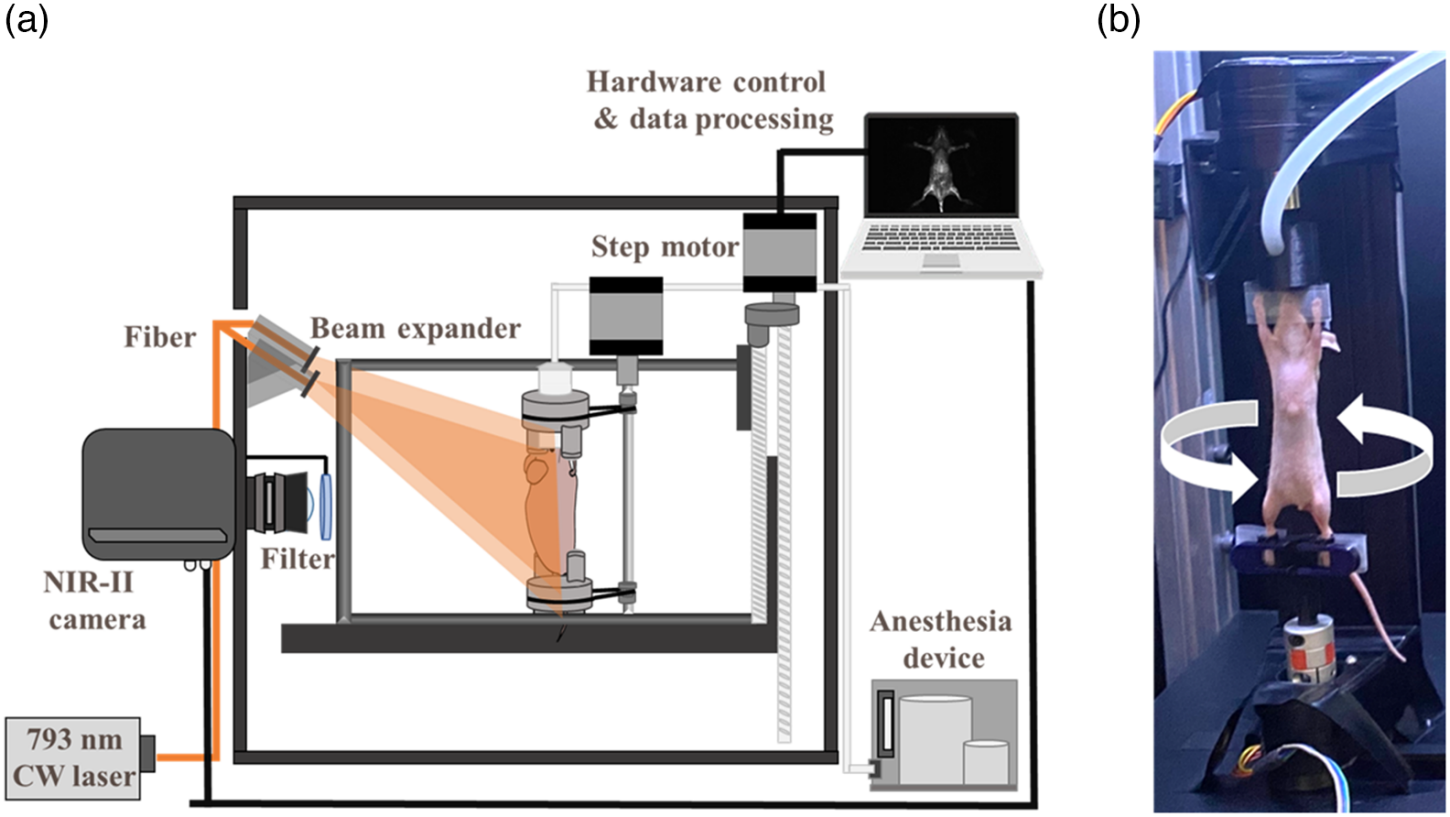
Home-built 3D NIR-II fluorescence rotational stereo imaging system: (a) schematic diagram of the system and (b) photograph of the experimental setup of *in vivo* optical imaging.

A schematic of a 3D NIR-II fluorescence rotational stereo imaging system is shown in Fig. 1.

The optical system includes an SWIR camera (InGaAs camera, NIRvana 640; 640×512  pixels, response 900 to 1700 nm) with a 35-mm C-mount zoom lens (LM35HC-SW, Kowa, Tokyo, Japan) and a 793-nm continuous-wave fiber laser (CNI laser, FC-W-793). The final measured optical spatial resolution results is given in Sec. S1 in the Supplementary Material. In air, the measured spatial resolution of the camera is consistent with the theoretical spatial resolution limit of 0.15 mm for the camera’s hardware (Sec. S2 in the Supplementary Material). A ground glass diffuser (Thorlabs DG10-220-MD) was utilized to homogenize the laser beam, creating a uniform light that illuminated objects. The laser power for absorption/fluorescence measures was $50\;\mathrm{mW}/{\mathrm{cm}}^{2}$. For blood vessel imaging, a 1300 long-pass (LP) filter (FELH1300, Thorlabs) and a 1000 LP filter were adopted to eliminate light interference below 1000 nm. We employed a 1300 LP filter (FELH1300, Thorlab) on the filter wheel to capture fluorescence above 1300 nm with lower scattering, as the longest emission tail of the NIR-II dye that we utilized could reach this range. Additionally, using a 1000 LP filter (MIDOPT LP1000) mounted on the lens, we were able to filter out scattered light and reduce background noise, thus achieving a high-contrast NIR-II fluorescence image. A 360-deg scanning module consisting of a mouse holder and a rotational stage was adopted to capture the NIR-II fluorescence stereo images of the mice. The homemade animal holders kept the mouse stable during the imaging process. Moreover, the fluorescence images obtained by 360 deg scanning and the 3D mouse’s contour were constructed by stacking different angles. The scanning angle of each section was ±3.5  deg from vertical.

### 2.2 NIR-II Fluorescence Rotational Stereo Imaging

**Fig. 2:**
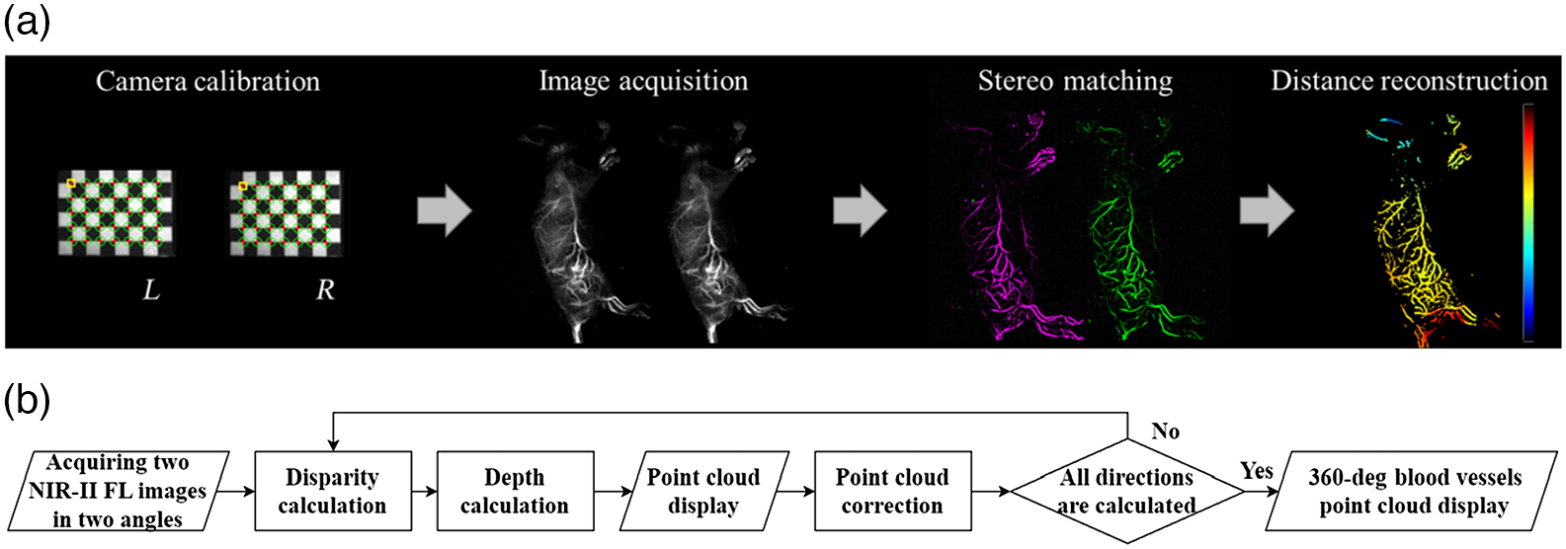
(a) Overview of the stereo imaging system for 3D vascular imaging. (b) The pipeline of the NIR-II fluorescence rotational stereo imaging method.

The rotational stereo vision imaging process is displayed in Fig. 2(a). The first step measured the camera parameters. The second step regulated the epipolar lines in the left and right eye views. Stereo matching is the key step in which pixel-wise correspondences were found between two eye views to generate a disparity map. A semi-global matching algorithm in MATLAB was used to calculate the disparity map. The final step reconstructed 3D information from the disparity map using the triangulation technique. For 3D contour imaging, we acquired 2D mouse contour images with different camera viewing angles of +θ and −θ under incandescent lamp illumination. We rotated the mouse and obtained 2D mouse contour images at different viewing angles (0 deg, 90 deg, 180 deg, and 270 deg). Finally, the 2D surface contour images were processed using MATLAB (MathWorks Inc., Natick, Massachusetts, United States) to obtain 3D contour images. Figure 2(b) shows the pipeline of our NIR-II fluorescence rotational stereo imaging. Both 3D vessels and contour imaging followed the same procedures for generating 3D images. Only one camera and a rotational stage were employed in building 3D vessel images from a left-eye view and a right-eye view. The different camera viewing angles of the objects result in displaced object locations on the images. The advantage of using a rotational stage instead of a moving plate is that the rotational stage has a larger stereo baseline. Thus the 3D whole-body vasculature images and contour images can be reconstructed with NIR-II rotational stereo vision.

**Fig. 3:**
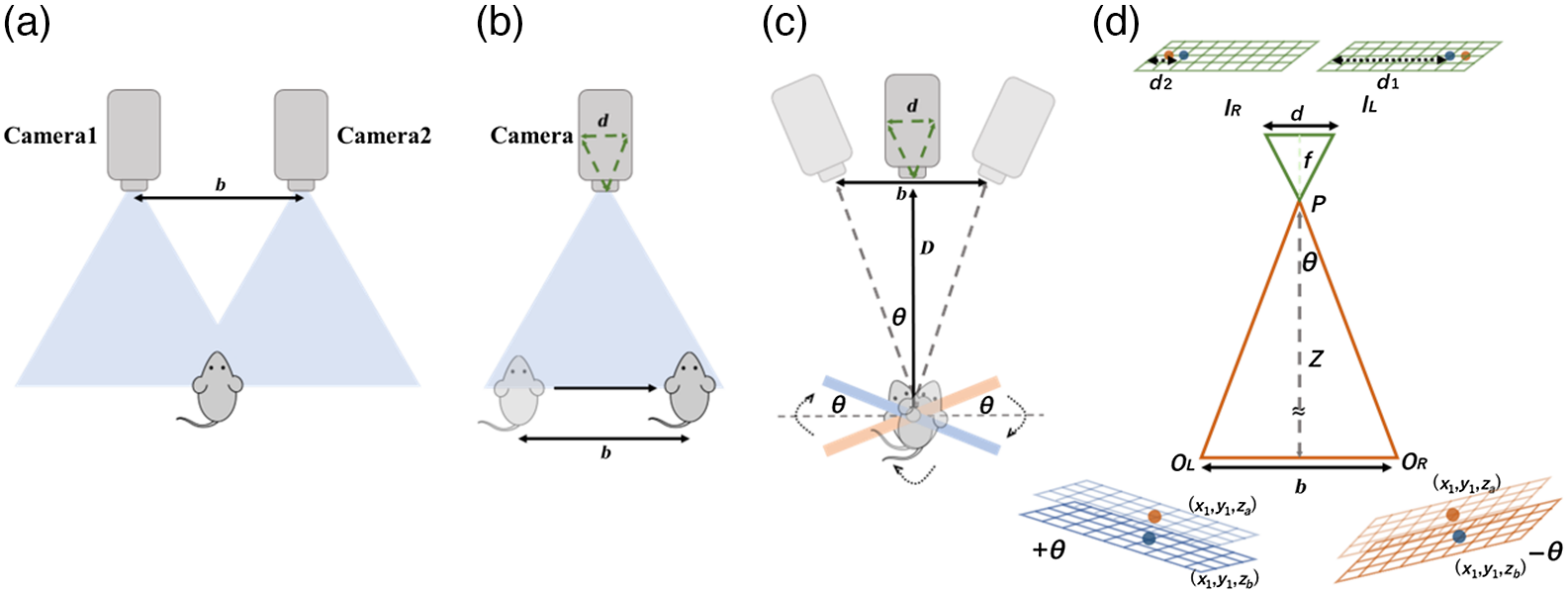
(a) Two-camera stereo system principle; (b) one-camera stereo system principle; (c) a top view of a rotational stereo vision setup; and (d) schematic of the rotational stereo vision imaging coordination system.

A two-camera stereo vision technique calculates the depth of objects by utilizing two cameras with a stereo baseline length of b to capture images of the same scene from two different perspectives [Fig. 3(a)]. A single-camera stereo vision technique captures two images of the same scene from slightly different angles, allowing for the calculation of the depths of objects [Fig. 3(b)]. The setup of the rotational stereo imaging is shown in Fig. 3(c). The rotational stereo imaging can be transformed into a standard stereovision problem. A rotational stereo technique captures two images of the same scene from slightly different rotational angles, thus enabling the calculation of the depths of objects present in the scene. A rotational angle was based on the baseline length and the distance between the camera and imaging plate, D. The different camera viewing angles of the objects cause a stereoscopic disparity in the sensor. The disparity is the displacement of a particular object between the left and right images. Figure 3(d) displays the distance measurement of the stereo imaging. The black and brown images are two images with different camera viewing angles +θ and −θ acquired from Fig. 3(c). The baseline b of the stereo coordinate system is the distance between the two eye-view images with their respective optical centers $O_{L}$ and $O_{R}$. The focal length f of a lens is the distance from the lens to the digital camera sensor and is much smaller than the object distance Z. Two eye-view images with optical centers $O_{L}$ and $O_{R}$ are separated by a baseline b. $I_{L}$ and $I_{R}$ are the image locations on the left and right eye views. We used the following stereo triangulation formula, which is based on a similar triangle theorem, to calculate the object distance Z in a stereo vision system:^36^

$$Z\approx \frac{b*f}{d},$$ (1)

where b is the stereo baseline, f is the focal length, and d is the disparity ($d_{i}=d_{j}-d_{k}$, j=1,3,5,…, k=2,4,6,…).^37^

The depth resolution $D_{r}$ is calculated with the triangulation Eq. (2) as

$$D_{r}\approx \frac{Z^{2}}{f*b}*\Delta p,$$ (2)

where Z is the distance from the object to the camera, b is the baseline length, f is the focal length of the camera, and Δp is the matching error in pixels (disparity values).^38^ At the distance between the camera and imaging plate, D of 440 mm, the matching error in pixels was 0.004 mm and the baseline length was 60 mm, resulting in a depth resolution of 0.3 mm.

### 2.3 Experimental Animals

The animal study protocol was approved by the Institutional Animal Care and Use Committee of the National Yang Ming Chiao Tung University (1100509, approved date May 9, 2021). BALB/c nude female mice (5 to 6 weeks, 18 to 22 g) were purchased from the National Laboratory Animal Center, Taipei, Taiwan. The mice were housed under normal laboratory conditions (22±2°C) and a regular 12 h light/dark cycle with free access to food and water. To transfer the tumor cells to the mice, a total of $1\times 10^{6}$ 4T1 cells with a volume of 200  μL were injected into the right hindlimb of BALB/c female nude mice subcutaneously.

### 2.4 Tumor Model

The 4T1 triple-negative murine breast cancer cell line was purchased from ATCC (American Type Culture Collection, Manassas, Virginia, United States). Cell lines were maintained in RPMI (GIBCO^®^ invitrogen Inc., Carlsbad, California, United States) medium with 10% fetal bovine serum (HyClone^®^ Thermo, Waltham, Massachusetts, United States), 50  μg/mL of penicillin/streptomycin (Sigma-Aldrich Co., St. Louis, Missouri, United States), and 2 mM of l-glutamine (Sigma-Aldrich Co., St. Louis, Missouri, United States) and were incubated at a 37°C in a humidified incubator with 5% ${\mathrm{CO}}_{2}$ and passaged every two days. The 4T1 mammary carcinoma was selected in this research due to its highly tumorigenic and invasive characteristics. In addition, the primary tumor could easily be surgically removed, which helps conduct an *ex vivo* tumor observation.

### 2.5 Fluorescent Agent

Our NIR-II imaging system can work with NIR-II fluorescent probes with emission peaks that are in the NIR-II region (1000 to 1700 nm), such as indocyanine green, IR-1061, IR-783, and IR-TPE Pdots. The NIR-II fluorescent agent, IR-TPE Pdots (MW: 2.4 kDa), which was applied in this research, was synthesized by the procedure of our recent study.^19^ The emission peak of the IR-TPE Pdot fluorescence spectrum is about 1000 nm and extends over 1200 nm, characterized by fluorescence spectroscopy when excited by a 793 nm NIR laser. The hydrodynamic diameter of the IR-TPE Pdot is about 45 nm, analyzed by dynamic light scattering. The imaging probe was administered at a dosage of 40 mg/kg body weight, and no adverse health effects were observed. All animals exhibited normal behavior and had normal health statuses throughout the study. This dose was selected to prevent signal saturation in the NIR-II fluorescence imaging while still allowing for adequate integration time. The mice were intravenously injected with IR-TPE Pdots (200  μL, 5  mg/mL) (n=3) for imaging at different time points. All mice for imaging were anesthetized with 2% isoflurane in air during the tail vein injection of the agent and image acquisition period.

## 3 Results

### 3.1 NIR-II 3D Rotational Stereo Imaging of Tumor-Bearing Mice

**Fig. 4:**
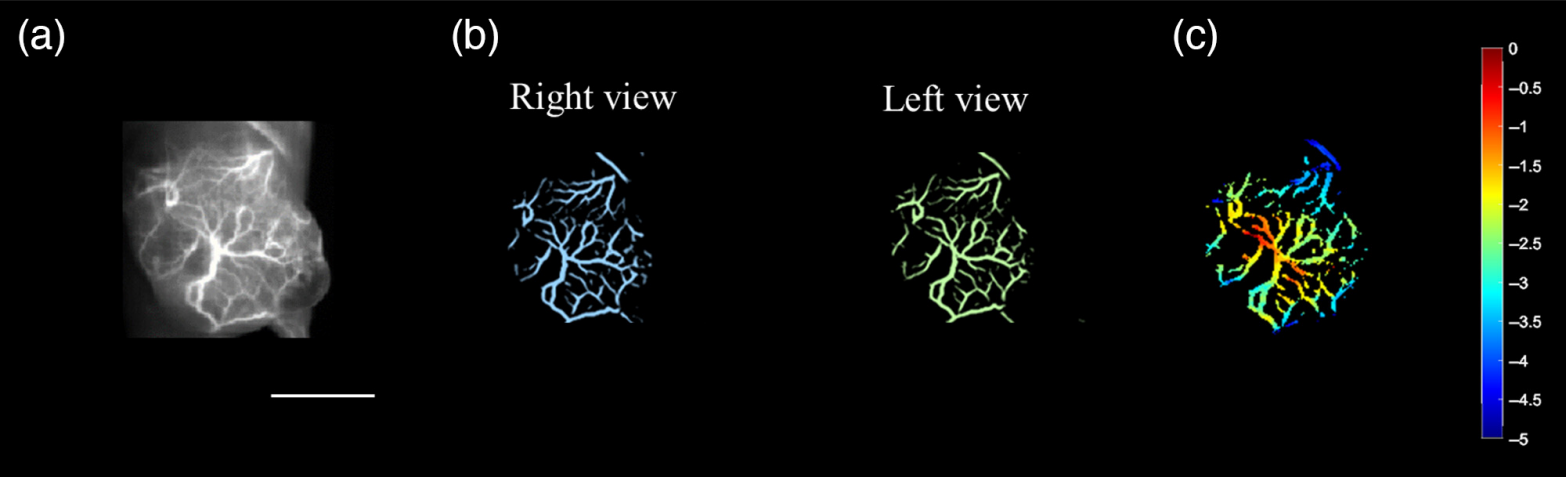
NIR-II rotational stereo image of a tumor blood vessel network: (a) NIR-II 2D image (scale bar: 10 mm); (b) two camera views; and (c) depth map of the blood vessels in the tumor region.

An *in vivo* test was performed to validate the feasibility of the system. A subcutaneous tumor was implanted in the right leg/hindlimb, and the experiment started when the diameter of the tumor reached 12 mm (n=3). An NIR-II fluorescence rotational stereo imaging system collected an NIR-II fluorescence image [Fig. 4(a)]. Then the system was used to acquire a left-eye camera view angle (green) and a right-eye view (blue), which resulted in displaced object locations on the images [Fig. 4(b)].In addition, we applied a vessel-enhancing algorithm based on the Hessian matrix to increase the image contrast, extract vascular structures, and filter non-vascular networks.^39^ A depth map [Fig. 4(c)] was then calculated from the disparity map according to Eq. (1). The result shows that the tumor vascular network (imaging depth 0 to −5  mm) was correctly reconstructed through system performance estimation. The different blood vessel depths are indicated with a color bar. In addition, the blood vessels were displayed in different colors for different depths in the tumor area of the mice.

### 3.2 NIR-II 3D Rotational Stereo Imaging of Whole-Body Vasculature

**Fig. 5:**
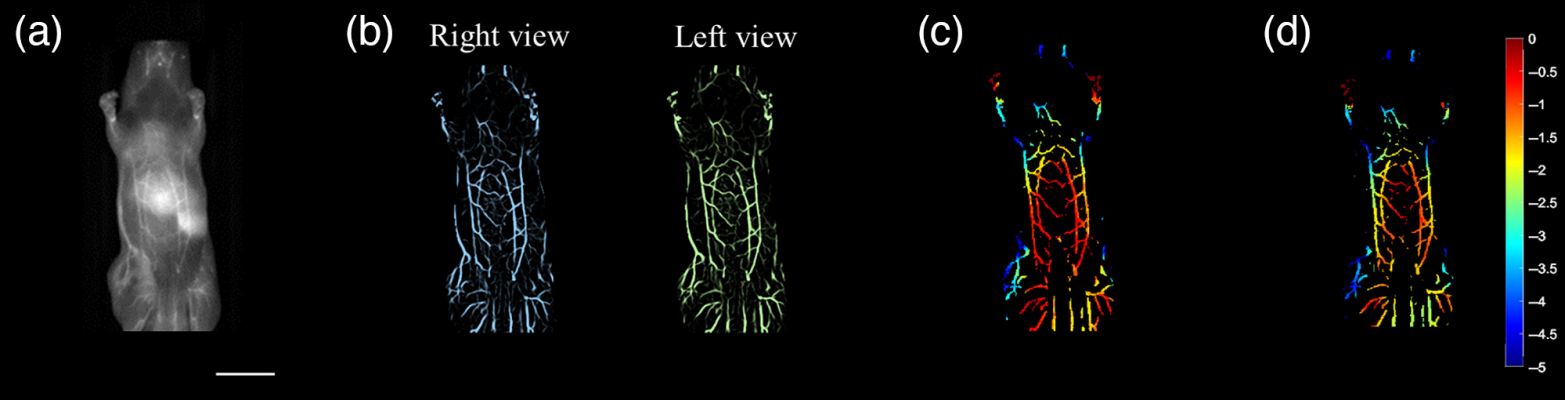
NIR-II rotational stereo image of a mouse’s abdominal region: (a) NIR-II 2D image (scale bar: 10 mm); (b) two camera views; (c) depth map of the abdominal blood vessels with a small viewing angle; and (d) depth map with a large viewing angle.

In this test, we performed blood vessel imaging of mice following intravenous injection of IR-TPE Pdots (5  mg/ml) (n=3). 2D fluorescence imaging of blood vessels was captured at 10 min post-injection with a 1300 nm LP filter [Fig. 5(a)]. We acquired a left-eye view (green) and a right-eye view (blue) with a customized NIR-II 3D rotational stereo imaging system with a rotation of 3.5 and 6 deg for 3D NIR-II imaging. The distance between the camera and imaging plate D of 440 mm. Figure 5(b) shows a left-eye camera view angle (green) and a right-eye view (blue). A disparity map can be turned into a depth map from two images taken from distinct camera viewpoints. Figures 5(c) and 5(d) show that the depth map of abdominal vessels (imaging depth 0 to −5  mm) was correctly reconstructed through system performance estimation. There are four main blood vessels with two layers. Major abdominal blood vessels were presented in multiple colors at different depths. Specifically, rotational stereo imaging with a larger stereo baseline length can distinguish the different layers of the blood vessels in the abdominal region.

### 3.3 NIR-II 3D 360-deg Contour Imaging of Phantom

**Fig. 6:**
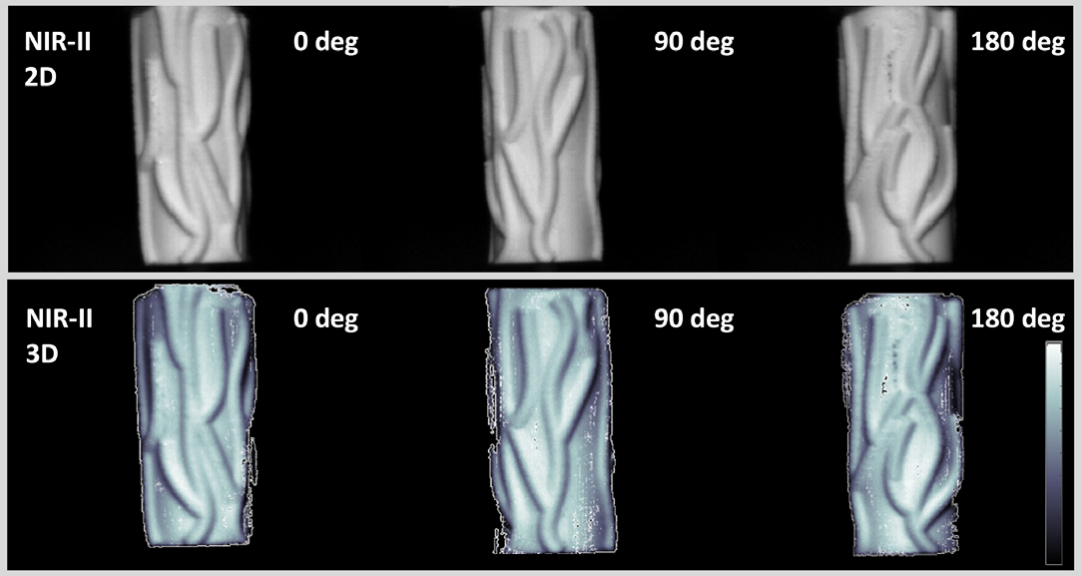
3D contour of a cylinder phantom with a blood vessel pattern using NIR-II rotational stereo imaging.

This section displays the reconstructed 3D point clouds of a cylinder phantom with vessel patterns. To validate the feasibility of the system, we performed experiments using an experimental phantom. The phantom was a circular cylinder with a radius of 20 mm and a height of 50 mm. The experiments were performed under incandescent lamp illumination, which could be detected by an NIR-II camera. Later, we used the imaging system to acquire left and right eye views with 8 camera-view angles, totaling 16 images. Each of the two left and right eye-view images were used to reconstruct a depth map. Then all of the depth maps with different viewing angles were transformed into a 3D model. The results of applying the proposed method are shown in Fig. 6. The first row shows the 2D NIR-II fluorescence images captured by a 1000 nm LP filter. The second row shows the 3D point cloud with a texture of different camera view angles (0 deg, 90 deg, and 180 deg). In other words, the rotational stereo imaging system can reconstruct the contour of the phantom.

### 3.4 NIR-II 3D Rotational Stereo Imaging of Mice Contour and Whole-Body Blood Vessels

**Fig. 7:**
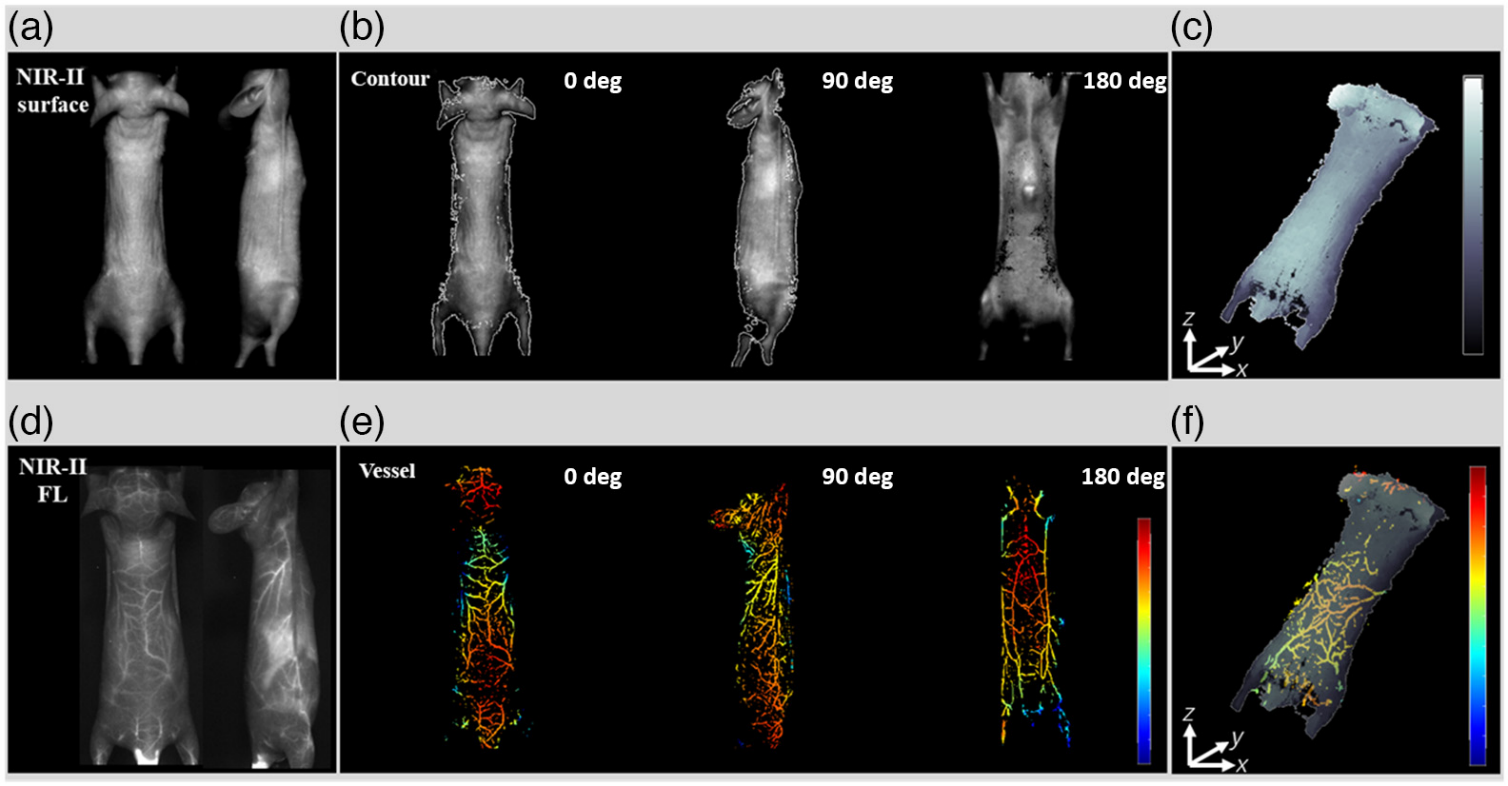
3D mice contour and whole-body vasculature using NIR-II rotational stereo imaging: (a) NIR-II surface images; (b) 3D object of mice contour with texture; (c) 3D depth map of contour; (d) NIR-II fluorescence blood vessel images; (e) 3D object of whole body blood vessels; and (f) 3D depth map of blood vessels and contour (Video 1, MP4, 2.93 MB [URL: https://doi.org/10.1117/1.JBO.28.9.094807.s1]).

Finally, we further experimented on nude mice in our 360-deg NIR-II rotational stereo imaging system. Mice were anesthetized with 2% isoflurane saturated with $O_{2}$ and were placed on a rotational stage. First, we acquired the 2D NIR-II fluorescence images of whole-body blood vessels under laser illumination. Second, the 2D NIR-II mice contour images were collected under incandescent lamp illumination. The 3D models were reconstructed from eight camera-view angles. Figures 7(a) and 7(d) show the obtained NIR-II 2D gray-level images and fluorescence images of the whole-body blood vasculature in mice. Figures 7(b) and 7(e) present the NIR-II images of the 3D model of the mouse contour with texture and the 3D whole body blood vessels in a color bar in different viewing angles (0 deg, 90 deg, and 180 deg). Figures 7(c) and 7(f) and Video 1 present the 3D depth map of mice contour and blood vessels in a color bar.

## 4 Discussion

This study pioneered the development of a 3D NIR-II fluorescence rotational stereo small animal imaging system (Fig. 1), boasting a 360-deg rotational stereo imaging technique, an NIR-II dye, and an NIR-II camera dedicated to small animal 3D NIR-II imaging. The NIR-II rotational stereo imaging system has a better depth resolution and a larger view angle than the device developed in a previous study.^35^ The performance of this system was quantified via the application of a rotational stereo imaging technique to successfully reconstruct a phantom, whole-body vasculature, tumor blood vessels, and mice contour.

The 3D NIR-II rotational stereo small animal imaging system has several advantages. (1) The setup of a rotational stage, in place of a moving plate, facilitates the acquisition of depth information and shortens scanning time. (2) The rotational stereo imaging system can reconstruct 360-deg whole-body vessel structures with a much wider viewing angle than the binocular stereo in-depth imaging system. (3) The system, furnished with an NIR-II camera and a rotational stage, is more cost-effective than other optical imaging techniques. (4) The custom-made ultra-bright fluorescent agent IR-TPE Pdots enables a sufficient penetration depth in blood vessel imaging. (5) The NIR-II rotational stereo technique enables sufficient resolution for the depth information, along with contour information, of the 3D vasculature image of nude mice.

This study reconstructed the depth maps of tumor vasculature and abdominal vessels of mice with the 360-deg rotational stereo method (Figs. 4 and 5), along with a rotational stage, as a substitute for a moving place, to improve the depth resolution with a larger stereo baseline. We used our vessel enhancement algorithm to filter out fluorescence signals below a threshold, resulting in high SNR images. Some vessels on the contour of the mouth were missed in the depth map. To rectify this, we adjusted the threshold selection to show different levels of blood vessel intensity with our vessel enhancement algorithm. As shown in Eq. (2), the depth resolution is positively proportionate to the square of the distance but inversely proportionate to the baseline length. Compared with our previous binocular stereo imaging system, the depth resolution of the proposed stereo imaging system was significantly improved by increasing the stereo baseline. In the proposed stereo imaging system, the distance between the camera and imaging plate D was 440 mm, and the baseline length was 60 mm, resulting in a depth resolution of 0.3 mm. In the previous binocular stereo imaging system, the distance between the camera and imaging plate D was 450 mm, and the baseline length was 40 mm, resulting in a depth resolution of 0.6 mm. The tumor vasculature image of mice shows that the blood vessels around the tumor site have different depths, with an in-depth range from −3.5 to −0.5  mm. The results of NIR-II stereo imaging were compared with those of photoacoustic imaging shown in Sec. S3 in the Supplementary Material. The rotational stereo method significantly improved the quality of reconstructed images due to the employment of a larger stereo baseline.

In the imaging process, a vascular enhancement and segmentation algorithm was used to improve the vessels’ image quality.^40^ The quality of the tumor vasculature’s depth map can be improved by extracting vessel information and filtering the object edge. Additionally, tumors larger than 1–2 mm in size can induce the development of new blood vessels for supplying nutrients, such as oxygen or glucose. Consequently, antiangiogenic drugs have been widely used to inhibit the development of these new tumor vessels. Overall, the 3D NIR-II rotational stereo vision technique is instrumental in visualizing 3D vessel structures and tumor vessels in small animals.

The study further validated the system’s capability in NIR-II contour imaging via 3D NIR-II reconstruction of a cylinder phantom (Fig. 6) and whole-body contour and vessel structures *in vivo* (Fig. 7). The NIR-II imaging system can reconstruct 3D contour images of small animals in the NIR-II band with incandescent lamp illumination. The 3D point cloud of the cylinder phantom was accurately presented from three different viewing angles (at 0 deg, 90 deg, and 180 deg). The 3D model provides further image depth information in displaying the location than the 2D image.^41^ In the 3D model, some stereo matching errors in the 3D model can be reduced by adding more viewing angles. Furthermore, in an *in vivo* experiment, the system produced the NIR-II 3D contour imaging of mice with incandescent lamp illumination (with >1000  nm) and whole-body blood vessel imaging with 793 nm laser fluorescence excitation. Our imaging system accurately co-registered the 3D blood vessels reconstruction result in the NIR-II region with 3D contour images under incandescent lamp illumination using the same coordinate system. By integrating the 3D contour and blood vessels, we can further display the depth map of the blood vessels under the skin. In addition, we can reconstruct tumor 3D contour with 3D blood vessel distribution surrounding or inside the tumor. Above all, the 3D NIR-II stereo vision technology can be instrumental in acquiring a 3D profile of the object’s contour. Furthermore, NIR-II imaging has already been used for clinical purposes.^42^^,^^43^ Our 3D NIR-II rotational stereo vision technique has the potential to be a highly valuable tool for 3D imaging in image-guided surgery. It is not only a powerful tool for assessing the tumor microenvironment, but it can also serve as an alternative to existing angiography techniques.

For the noticeable results of the study, further improvement could be achieved via the following modifications. First, given the difficulty in detecting deeper vasculature structures in the NIR-II window, the current imaging depth could be increased further to be larger than 5 mm by extending the absorption and emission of the fluorescent agents to the NIR-IIb (1500 to 1700 nm) region. Second, for better performance of the imaging system, the depth resolution can be improved by enlarging the stereo baseline or increasing the lateral resolution using camera lenses with a longer focal length. The third is the optimization of the vessel enhancement algorithms for vascular extraction and quantification, such as the size of arterial and venous vessels. Fourth is the qualification of vessel density with 2D NIR-II fluorescence images and 3D blood vessel structure. Then we could estimate the effect of the drug on inhibiting angiogenesis.

## 5 Conclusion

We developed an innovative 3D NIR-II rotational stereo imaging system, which was applied in small animal 3D NIR-II fluorescence blood vessels imaging, tumor imaging, and contour imaging. The 360-deg rotational stereo version achieved a 0.3 mm depth resolution, a 2× improvement in depth resolution over the traditional two-view stereo method. In summary, we have explored the benefits of this 3D imaging system for the realization of 360-deg whole-body contour and tumor blood vessels imaging using one NIR-II camera. Future work would extend the application to NIR-IIb deep vasculature imaging for small animals.

## Code, Data, and Materials Availability

The data used in this paper are available upon request.
