# Supplementary material for: Realization of NIR-II 3D whole-body contour and tumor blood vessels imaging in small animals using rotational stereo vision technique
Source: J Biomed Opt. 2023 May 24;28(9):094807. doi: 10.1117/1.JBO.28.9.094807 (PMC10208585; doi:10.1117/1.JBO.28.9.094807)
Supplement: Supplementary file 1 [file JBO_028_094807_SD001.pdf]

Supplemental Material for Realization of NIR-II 3D whole-body contour and tumor blood vessels imaging in small animals using rotational stereo vision technique

S1 Optical Spatial Resolution

The 1951 USAF resolution chart, a standard in optical spatial resolution measurement, was used as the optical spatial resolution phantom. This phantom was mounted onto the imaging platform of our NIR-II fluorescence imaging system, and the objective was finely adjusted so that the phantom was in focus with a fully open aperture. Following this, the camera's vertical and horizontal optical spatial resolution was measured in air. From the 1951 USAF resolution chart provided by Yoshihiko Takinami, the Group 6, Element 6 matches the Group 1, Element 6, 1951 USAF resolution test chart with MIL-STD-150A format, R=3.56 (lp/mm)

$$R = \frac{1000\ \mu\text{m}}{2 \times 3.56} = 140\ um = 0.14\ mm$$

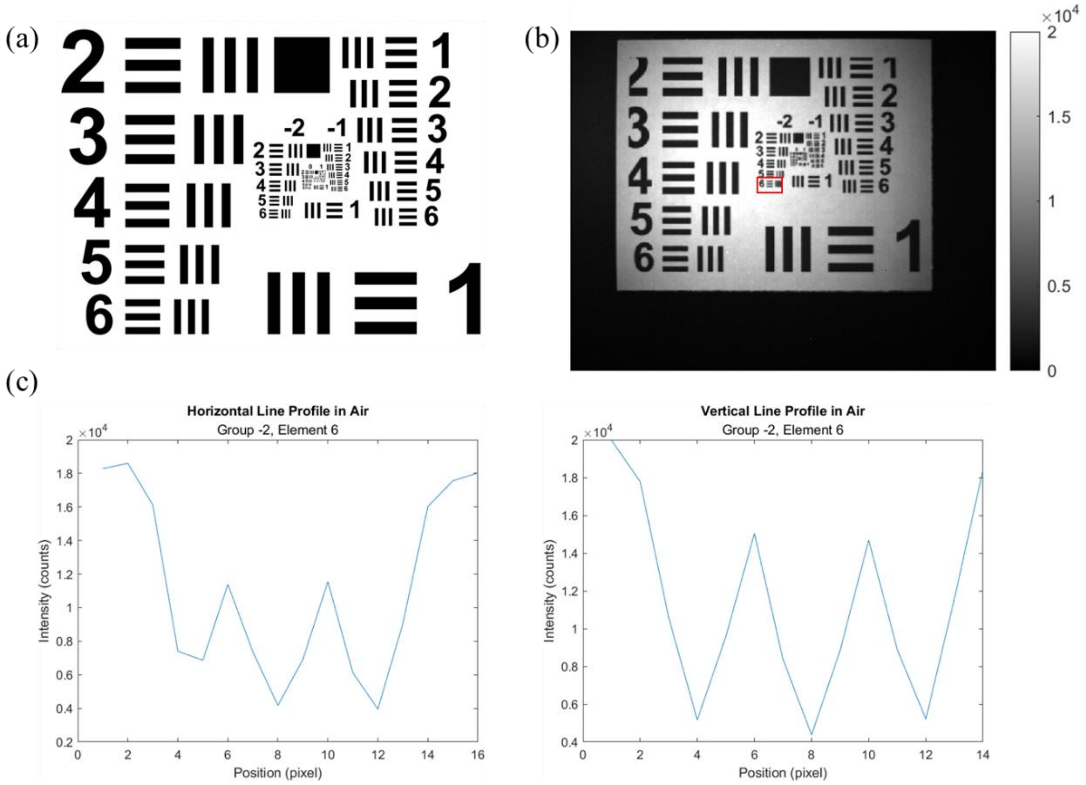

Figure S1 Images of the optical resolution phantom: (a) a 1951 USAF resolution test chart, (b) inside the imaging chamber with air, (c) the extracted line profiles from the optical spatial resolution test chart's smallest resolvable element.

S2 Spatial Resolution Limit of Optical Detector

Below, we list relevant parameters and calculate the spatial resolution limit of our optical detector. Our NIR-II fluorescence imaging system features an optical detection channel with a field-of-view of 96 x 76.8 mm^2. This calculation provides the theoretical limit of optical spatial resolution detection based on the hardware used.

NIR-II fluorescence imaging system parameters:
Pixel Size (s): 20 μm x 20 μm
Number of Pixels (p): 640 x 512
Image Field of View (FoV): 96 x 76.8 mm^2

Limiting sensor resolution:

$$R_I = \frac{1}{2 \times s} = \frac{1}{2 \times 20\ \mu\text{m}} \times \frac{1000\ \mu\text{m}}{1\ mm} = 25\ \frac{lp}{mm}$$

Where RI is the image space resolution.

Sensor dimensions:

$$S_H = s \times p = 20\ \mu\text{m} \times 640 \times \frac{1\ \text{mm}}{1000\ \mu\text{m}} = 12.8\ mm$$

$$S_V = s \times p = 20\ \mu\text{m} \times 512 \times \frac{1\ \text{mm}}{1000\ \mu\text{m}} = 10.24\ mm$$

Where subscript H stands for horizontal and subscript V stands for vertical.
Magnification:

$$m = \frac{\max\,(S_H, S_V)}{\text{FoV}} = \frac{12.8\ mm}{96\ mm} = 0.133\ X$$

Spatial resolution limit:

$$R = R_I \times m = 25\ \frac{lp}{mm} \times 0.133 = 3.325\ \frac{lp}{mm}$$

$$R = \frac{1000\ \mu\text{m}}{2 \times 3.325} = 150\ um = 0.15\ mm$$

Where R is the optical spatial resolution limit of the system.

S3 Depth comparison of NIR-II fluorescence imaging of blood vessels and photoacoustic images of blood vessels

We compared NIR-II fluorescence images of blood vessels and photoacoustic (PA) images of blood vessels. A Q-switch Nd YAG laser at 532 nm and a commercial transducer (A313S, OLYMPUS NDT) with a center frequency of 15 MHz have been integrated into our newly developed PA imaging system. For imaging the abdominal vessels, there were four main blood vessels. The results of the PA image showed that the distance between the first and second blood vessels was 0.2 mm. The results of NIR-II fluorescence images showed that the distance between the two blood vessels was 0.3 mm (orange and yellow color). Moreover, in the PA image, the distance between the third and fourth blood vessels was 1 mm. The results of the NIR-II fluorescence image of the abdominal vessels showed that the distance between the two blood vessels was 0.7 mm (orange and yellow color). All the detailed information is shown in Figure S3 and Table S1.

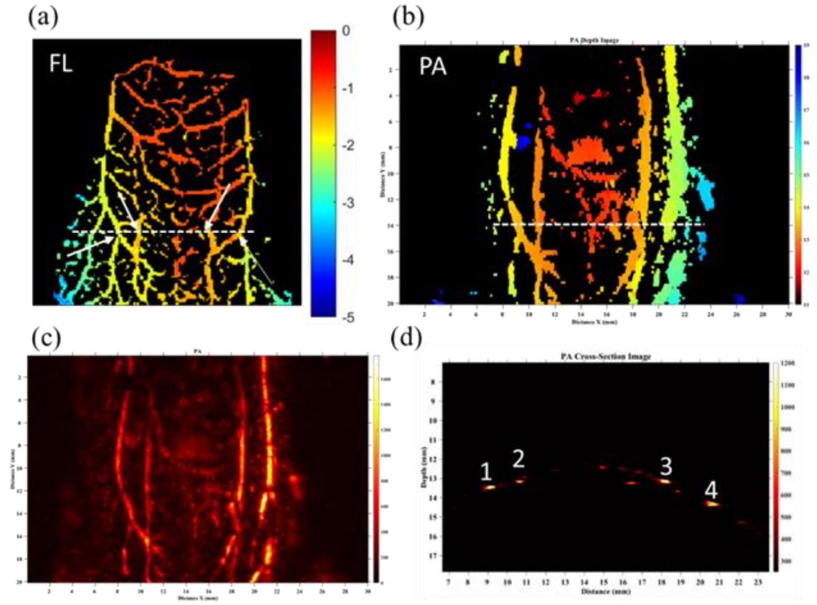

Figure S3 (a) NIR-II fluorescence depth map of the blood vessels in a mouse's abdominal region (b) Photoacoustic image of a mouse's abdominal region (c) Reconstructed maximum intensity projection (MIP) of the photoacoustic image in the x-y direction (d) Photoacoustic section image of a mouse's abdominal region.

Blood vessels	**1**	**2**	**3**	**4**
FL depth (mm)	0	+0.3	+0.7	0
PA depth (mm)	0	+0.2	+0.3	-0.7

Table S1 Depth information for blood vessels in a mice's abdominal region: Above, we present a table of the depth information. The values shown here correspond to a depth of 0 mm for the first blood vessel in Figure S3.
